# Supplementary material for: Discovery of a Potent PLK1-PBD Small-Molecule Inhibitor as an Anticancer Drug Candidate through Structure-Based Design
Source: Molecules. 2019 Nov 28;24(23):4351. doi: 10.3390/molecules24234351 (PMC6930574; doi:10.3390/molecules24234351)
Supplement: Supplementary file 1 [file molecules-24-04351-s001.pdf]

# Supplementary Materials for

**Discovery of a potent PLK1-PBD small-molecule inhibitor as an anticancer drug candidate through structure-based design**

Yunjiang Zhou[1,2], Fang Yan[1], Xiangyun Huo[1] and Miao-Miao Niu[1,*]

[1]*Department of Pharmaceutical Analysis, China Pharmaceutical University, Nanjing 210009, China*

[2]*State Key Laboratory of Natural Medicines, School of Basic Medicine and Clinical Pharmacy, China Pharmaceutical University, Nanjing 210009, China*

**Correspondence: niumm@cpu.edu.cn (M.-M.N.)*

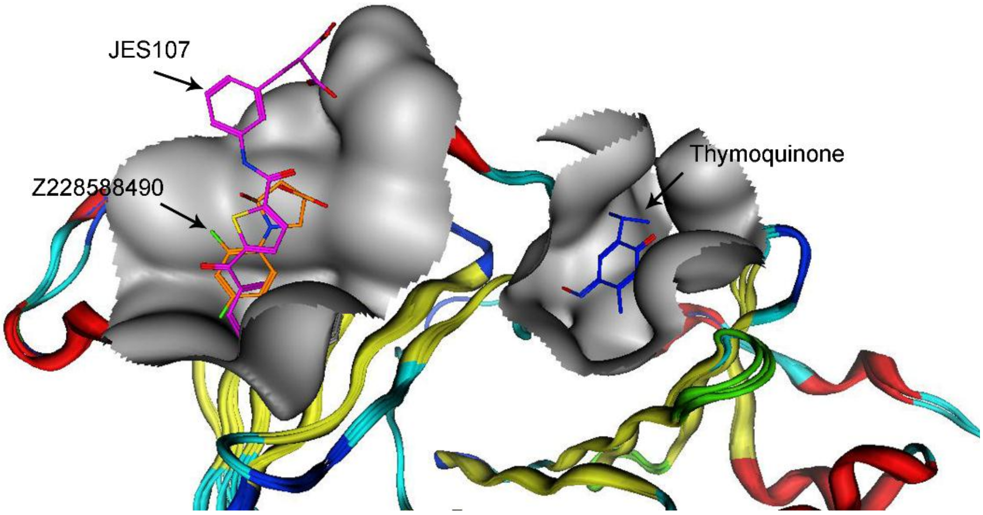


**Figure S1.** The overlay of three crystal structures (PDB code: 5NN2, 5NEI and 4H71). Binding pockets of PLK1-PBD as revealed by small-molecule ligands are shown in gray. The two crystal structures (PDB code: 5NN2 and 5NEI) of PLK1-PBD in complex with two ligands (Z228588490 and JES107) bound to the hydrophobic pocket (on the left side).The crystal structure (PDB code: 4H71) of PLK1-PBD in complex with a ligand (thymoquinone) bound to the positively charged binding pocket (on the right side). Z228588490, JES107 and thymoquinone are shown in sticks of orange, purple and blue, respectively.

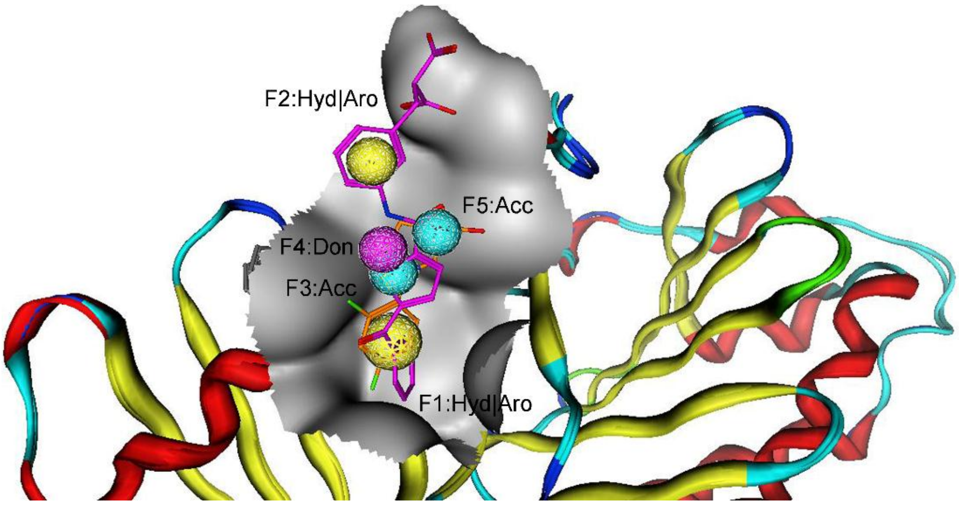


**Figure S2**. Binding pockets of the PLK1-PBD crystal structures. The binding pocket is shown in gray. Pharmacophore features are color-coded: Yellow, two hydrophobic and aromatic features (F1 and F2: Hyd|Aro); cyan, two hydrogen bond acceptor features (F3 and F5: Acc); purple, one hydrogen bond donor feature (F4: Don).

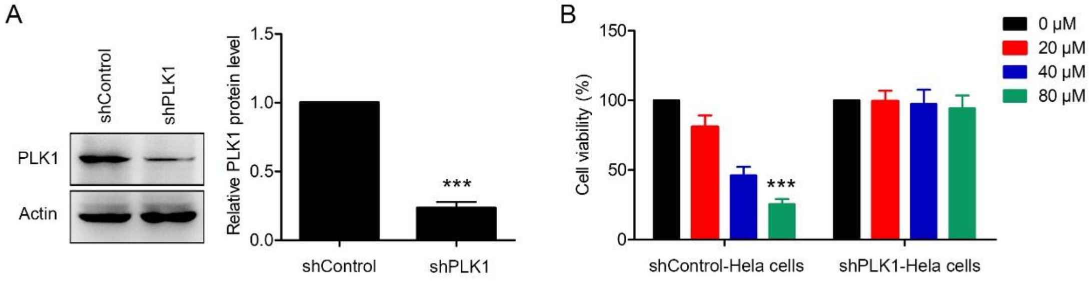


**Figure S3.** Target validation of hit-5 through the silencing of PLK1 gene by shRNA. (A) Western blot analysis of PLK1 protein levels in HeLa cells stably expressing Control shRNA and PLK1 shRNA. (B) The sensitivity of HeLa cells stably expressing indicated shRNA to hit-5 detected by MTT assay. shControl-HeLa cells: HeLa cells stably expressing Control shRNA, shPLK1-HeLa cells: HeLa cells stably expressing PLK1 shRNA. The results are representative of three independent experiments and are expressed as mean ± SD. ***$P < 0.001$.
